# EXPERTS II - How are patient and caregiver participation in health and social care shaped by experienced burden of treatment and social inequalities? Protocol for a qualitative synthesis.

**DOI:** 10.3310/nihropenres.13411.1

**Published:** 2023-06-16

**Authors:** Carl R May, Carolyn A Chew-Graham, Katie I Gallacher, Katja C Gravenhorst, Frances S Mair, Ellen Nolte, Alison Richardson

**Affiliations:** 1 Department of Health Services Research and Policy, London School of Hygiene & Tropical Medicine, London, UK; 2 NIHR ARC North Thames, London, UK; 3 School of Medicine, Keele University, Keele, UK; 4 School of Health and Wellbeing, University of Glasgow, Glasgow, UK; 5 Faculty of Health Sciences, University of Southampton, Southampton, UK; 6 NIHR ARC Wessex, Southampton, UK

**Keywords:** Burden of treatment, Qualitative evidence synthesis, Illness trajectories, Social inequalities

## Abstract

**Background:**

The workload health and social care service users and caregivers take on, and their capacity to do this work is important. It may play a key part in shaping the implementation of innovations in health service delivery and organisation; the utilisation and satisfaction with services; and the outcomes of care. Previous research has often focused on experiences of a narrow range of long-term conditions, and on factors that shape adherence to self-care regimes.

**Aims:**

With the aim of deriving policy and practice implications for service redesign, this evidence synthesis will extend our understanding of service user and caregiver workload and capacity by comparing how they are revealed in qualitative studies of lived experience of three kinds of illness trajectories: long-term conditions associated with significant disability (Parkinson’s disease, schizophrenia); serious relapsing remitting disease (Inflammatory Bowel Disease, bipolar disorder); and rapidly progressing acute disease (brain cancer, early onset dementia).

**Methods:**

We will review and synthesise qualitative studies of lived experience of participation in health and social care that are shaped by interactions between experienced treatment burdens, social inequalities and illness trajectories. The review will involve:

1.  Construction of a theory-informed coding manual; systematic search of bibliographic databases to identify, screen and quality assess full-text papers.

2.  Analysis of papers using manual coding techniques, and text mining software; construction of taxonomies of service user and caregiver work and capacity.

3.  Designing a model of core components and identifying common factors across conditions, trajectories, and contexts.

4.  Work with practitioners, and a Patient and Public Involvement (PPI) group, to explore the validity of the models produced; to develop workload reduction strategies; and to consider person-centred service design.

**Dissemination:**

We will promote workload reduction models to support service users and caregivers and produce policy briefs and peer-reviewed publications for practitioners, policy-makers, and researchers.

## Plain language summary

Our experiences of illness are often complex. We may have to work hard too. We may need to monitor and record symptoms: take up different diets and physical activity; use different drugs and medical devices; develop expertise in using websites and information technology; coordinate input from health and care services; sometimes we have to work out how to pay for the services we need.

How we get through this work is affected by our capacity to do it, and that is shaped by personal and wider resources, we can draw on. All of this is also affected by the services that are available to us, and by the ways our chances in life are shaped by income, ethnicity, education, gender, and age. The kinds of illnesses we have and how they progress, mean that these factors change over time. We call these changes trajectories.

To better understand service user work and capacity, we will review published studies that tell us about people’s everyday experiences of living with illnesses. We focus on three rarely studied trajectories. These are long-term conditions associated with significant disability; serious relapsing remitting disease; and rapidly progressing acute disease.

We will first use existing research to build a framework in which we can describe and understand relevant aspects of the published studies. We will use this framework to extract relevant information from the studies. This will enable us to make a model of common features of service user work and capacity across different conditions, their trajectories, service organisation and delivery, and patterns of social and economic disadvantage. Finally, we will work with groups of service users and caregivers, and with health and social care professionals to apply the model to the development of strategies to reduce workload and improve service design for people with complex health problems.

## Introduction

The workload that service users and caregivers take on, and their capacity to do this work, when they engage with and participate in different kinds of care is important and may play a key part in shaping the adoption and implementation of innovations in service delivery and organisation, utilisation and satisfaction with services, and the outcomes of care ^1^. This is reflected in policy and practice interventions that identify service users and caregivers as part of a team that consists of informal networks beyond provider organisations in health and social care, as well as professionals within them ^2^. Much work in this field has been aimed at service user and caregiver experiences of a narrow range of complex long-term conditions, and these studies have often focused on adherence to self-care regimes ^3^. In this review we will extend our understanding of service user and caregiver workload and capacity by comparing the ways that they are revealed in qualitative studies of the lived experience of a wider variety of physical and mental health problems characterised by long-term, relapsing remitting, and rapidly progressing trajectories. Using both conventional models of qualitative analysis and novel text mining approaches, we will explore the ways in which these experiences are shaped by interactions with self-care, healthcare, and social care professionals and provider organisations; by patterns of service organisation and delivery; and by different kinds of inequalities of access and provision of care, along with wider social inequalities. This evidence synthesis will create a taxonomy of service user and caregiver work associated with lived experiences of burden of treatment; a taxonomy of theoretical constructs that explain interactions between them; and identification of core components of service user and caregiver experience of configurations of care. These will support development and implementation of new, service user-centred models of care. 

## Background and rationale

### Service user and caregiver work

Over five decades, empirical research in medical sociology and social psychology, health psychology, and medical anthropology has led to a very large body of literature that points to the importance of service user and caregiver work in shaping engagement, participation, survivorship, and clinical and social outcomes across a range of healthcare problems ^1^. This corpus of studies has drawn attention to work that is intimately linked to being (negotiating experience and identity) ^4^; changing (managing status passage and biographical disruption) ^5, 6^; relating (participating in interactions within healthcare provision and informal social networks) ^7^; and doing (performing health behaviours and enacting healthcare technologies and self-care practices) ^8^. The work that service users and caregivers do has always been important but is becoming more so because healthcare provider organisations are increasingly promoting models of care in which service users and caregivers are seen to be integral to the healthcare team and thus the workforce.

This review focuses on service user and caregiver work and capacity, across a range of complex disease types and trajectories. Its main focus is on burden of treatment and the ways that it shapes the experience of service users and caregivers.

***Definition of participants***

Any study that investigates the experiences of people who use multiple services faces the problem of acknowledging and supporting the complex ways that they self-identify. People who are involved in self-care, or who are in remission from relapsing remitting diseases, may not see themselves as service users. People using formal health services may be called service users, but people who use mental health services, or who have recovered from other serious illnesses, may call themselves survivors. People who identify with the disability movement may want to use terminology that emphasises control. People using social care services may be called customers, clients or service users, but may self-identify in very different ways. In this proposal, but not in the review itself, we use the term service user. We use service user to mean someone who is sick, who is in a relationship with one or more health or social care services because of that sickness, and whose experience of that service is shaped by social inequalities of some kind. The term caregivers may refer to partners and spouses, other family members, friends, employees of service provider organisations, and even holders of power of attorney, official guardians and other officers of the court. The important feature of a caregiver is that they perform affective, cognitive, informational and material work with and for service users.

***Definition of service user and caregiver workload***

Service users and caregivers take on multiple tasks when they participate in care. These tasks include those that arise from negotiated obligations to participate in delegated clinical work, such as conforming to expectations of behaviour modification and change; symptom monitoring and management; adhering to complex treatment regimens and managing multiple drugs, dressings, medical devices, web-enabled tools and information sources, and prostheses. Beyond these negotiated obligations to perform clinical work that is handed off to service users and caregivers, are the assumed obligations that arise when service users and caregivers have to take on the organisational work that they need to do to engage, and stay engaged, with health and social care providers. This includes tasks relating to participation, accessing, navigating, coordinating and managing processes of care with (often uncoordinated) multiple service providers and their complex administrative systems and care pathways ^9– 11^.

***Definition of service user and caregiver capacity***

Service users and caregivers have finite capacity. We define capacity as the combination of affective, cognitive, relational, informational, material and economic resources available to the service user that make it possible for them to participate in care and to meet the normative expectations of provider organisations and professionals ^11^. Capacity is not just a function of individual characteristics of service users and caregivers, but is shaped by the *social and relational contexts* in which they are located. They experience structural social advantage and disadvantage; varying access to supportive social networks and social capital ^12^; and in their immediate social relations they draw on the collective resilience ^13^, competence ^1^, and efficacy ^14^ of network members. Capacity is also shaped by the pathophysiological, psychological, cognitive and emotional effects of disease as these play out over time ^15^; by social and institutional responses to particular forms of ill-health that include changes in social status, stigma, and assessments of culpability and the legitimacy of different expressions of symptoms.

***Definition of trajectories***

Health and social care services increasingly characterise the ways that people move through them and access care using the language of processes and pathways. Clinical pathways are system-level tools for organising service users according to diagnosis, treatment modality, professional contact, and disease progression. Pathways differ between different specialisms and healthcare provider organisations ^16^. However, they may not be configured or understood in the same way by social care providers. For service users and caregivers, the situation may be more complex ^17^. Disease progression itself may constitute a temporal trajectory. In some diseases, trajectories may run over many years and be associated with cumulative and often significant disability, as in Parkinson’s disease. In relapsing-remitting diseases like bipolar disorder trajectories may oscillate between recovery and recurrence and the future is characterised by uncertainty. In rapidly progressive acute diseases, such as some brain cancers, trajectories involve significant and often acute pathophysiological deterioration characterised by rapid loss of physical and mental capacity and struggles over rescue and recovery. These trajectories are more than changes that take place over time, and they are often more than the sum of pathophysiological processes. Instead, they may take the form of status passages ^5^, in which service users and caregivers’ social identities are formed and changed according to the ways in which others relate to the character and effects of their illness, the degree of disruption to relationships and socio-economic status that follow from it, and anticipated outcomes of disease progression ^6^.

### Sources of fragmentation in service provision

Interest in the conduct of service user and caregiver work reflects the ways that its character has changed as healthcare providers around the world have had to respond to an epidemiological transition from acute and infectious disease, to long-term, non-communicable, and comorbid conditions ^18^. These conditions are often associated with ageing. They are exacerbated by social inequalities, and there is good evidence that economic and educational inequalities are associated with earlier onset of comorbid long-term conditions ^19^. In response to these problems, innovations in the organisation and delivery of care have increasingly shifted locus of clinical activity away from individualised interactions between doctors and service users characterised by continuity of care, to transactional models of care in which service users encounter multiple service providers who perform specific technical tasks and who are located in complex technical divisions of labour ^20^. It is not clear whether the same situation applies in social care, but as service commissioners and providers have come under pressure to reduce costs and outsource services considerable fragmentation in service organisation and delivery has become evident. Service users and caregivers may thus be located in mixed economies of care provision that includes NHS, statutory social care, private sector, and third sector providers.

### Structural disadvantage affects participation

Complexity in experiences of health and social care, and of health and social care systems needs also to be understood in the context of well-established effects of socio-economic disadvantage, along with other structural disadvantages formed around gender, ethnicity, age, and migration. The role of structural inequalities in forming a context for sometimes hard and heavy work for service users and caregivers is well established ^21, 22^. Groups exhibiting structural advantages experience better health; fewer comorbidities; and later onset of chronic comorbidities. Importantly, observed interactions between advantaged populations and health services are characterised by experiences of better quality and easier access to healthcare, better access to formal and informal support mechanisms, and fewer environmental stressors ^23^. The health and social care landscape is shaped by unevenly distributed structural advantage, system-level forces, and epidemiological changes. These profoundly affect expectations of service user and caregiver contributions to their care; indeed they may experience extremes of disadvantage ^24^.

### Expectations of service users and caregivers

Changes in the character of service user and caregiver work in healthcare call for a radical reconsideration of their roles: indeed, policy and research initiatives link engagement in these processes of care to explicit expectations of participation in a healthcare ‘team’ or informal membership of the healthcare ‘workforce’ ^2^. These expectations of participation call for very different investments by service users and caregivers, in which individual motivation and adherence to treatment regimens are likely profoundly influenced by the workload that stem from them and their capacity to meet its demands ^15^. These investments are sometimes contested by service users and caregivers ^25^. These can require significant numeracy and literacy, as well as high level administrative and technological skills, in vulnerable and disadvantaged populations ^26^. This work is often distributed within complex social networks and relational processes ^18^. It thus calls for collective action, efficacy, and competence over and above individual psychological variables such as self-efficacy ^25, 27– 31^. It is not clear how these expectations and interactions correspond to those of social care, but it is likely that these are also unevenly distributed, and this unevenness may give rise to important inequalities in capacity, participation, and outcomes ^32^.

### Work leading up to this protocol

Following on from the germinal research presented in the ‘Background’ section, we have contributed to this literature. We have pointed to the importance of the workload that service users and caregivers take on when they have to manage their health and healthcare ^33^, and when they have to understand and organise their interactions with healthcare agencies and other entities ^34^. We have explored how self-care and healthcare workload can burden service users and their families, and how capacity to handle this workload varies between individuals. Our contributions to this have been through theoretical development as well as empirical research. Building normalization process theory has helped us understand the ways in which experiences of service user-hood can be understood as material and relational work that moves back and forth between the clinic and home; the cumulative complexity model, has helped us to understand service user workload and capacity over linear time and proposed that it is associated with poor healthcare utilisation and outcomes; and burden of treatment theory has helped us to understand the distribution of service user and caregiver workload and capacity over relational and organisational space ^1^. These developments enable us to model burden of treatment as a result of micro-level phenomena in which material and interactional practices are allocated and negotiated in complex interactions between people, disease processes, and healthcare environments ^10, 32, 35– 37^. Understanding service user capacity, balancing preferences, and controlling workload allocation and capacity will support the design of minimally disruptive models of care that work across sectors.

## Why is this research needed now?

### Why do we need another systematic literature review?

Much is now known about experiences of treatment burden in specific long-term conditions (especially diabetes, heart failure, chronic obstructive pulmonary disease (COPD), chronic kidney disease and stroke) because they are common diseases that generate high levels of demand, consequent workload and expenditure, and are targets for self-care interventions intended to promote service user activation and slow down disease progression. This had made them important foci for research. But focusing on conditions characterised by trajectories of disease progression and degeneration over several years means that important features of other kinds of illness are less visible. It is clear, for example, that experiences of symptoms and care, workload and capacity, are very different in disease of long duration (e.g. COPD) and relatively rapid progression (e.g. lung cancer) although these diseases have similar effects and are equally lethal ^38^. Much less is known about the ways that workload and capacity are constituted and experienced in mental health problems.

### Is a review of multiple disease types and trajectories, service contexts, and social contexts feasible?

To build more person-centred and responsive services we need to understand interactions between (a) service user and caregiver experiences; (b) service organisation and delivery; and (c) structural and system-level patterning of advantage. Although complex comparative qualitative syntheses are challenging to perform, we have previously demonstrated that such an approach is feasible and rewarding. In the EXPERTS I review ^32, 39^ we analysed and compared reviews of qualitative studies of lived experience of heart failure, COPD, and chronic kidney disease. This showed that the factors we wish to address are important. However, that review focused on the impact of factors related to management of workload and capacity in conditions marked by significant pathophysiological deterioration towards the end of life.

### The need for comparative analyses

Our systematic review of qualitative studies of experiences of self-care, health and social care is an important step towards modelling interactions between services and their contexts, developing instrumentation, and developing and evaluating interventions at the individual and organisational levels that will support service users and caregivers, and that will support demand management strategies at a system level. The review will focus on disease types and trajectories rather than single index conditions: we are interested in the ways that work and capacity are played out differently across a space characterised by different service providers and different patterns of social inequality, rather than by a specific clinical problem.

***Understanding service user experience beyond single index conditions***

Our comparator conditions are physical and mental health problems that are defined by one of three trajectories. These are: long-term conditions associated with significant disability, relapsing remitting disease, and rapidly progressive acute disease.

***Parity between condition types***

The review gives parity to physical and mental health problems, while recognising that these are experienced in different ways. For example, experienced workload and capacity are likely to be very different in schizophrenia and astrocytoma, in part because of differences in service organisation, service delivery, and social context.

***Parity between condition trajectories***

Most work on service user work, workload, and capacity has focused on conditions of relatively long duration in which behaviour modification and self-care are important components of management. Much less is known about relapsing-remitting conditions and the different workload and capacity problems that stem from them, or about diseases that progress rapidly to conclusion, in which workload and capacity may be transferred from service users to caregivers quite early in their trajectory.

***Multiple focal points***

Comparative analyses will reveal a core set of constructs which vary between index conditions, service contexts, and disease trajectories. They will also reveal the important differences in the ways in which workload and capacity are experienced across index conditions, three disease trajectories, and three service contexts.

## Aims and objectives

### Aim

We will review, compare, and synthesise qualitative studies of the lived experience of physical and mental health problems characterised by long-term, relapsing remitting, and rapidly progressing trajectories. In these contexts, we will (a) investigate the work of service user and caregiver engagement and participation in self-care, health and social care; (b) understand how these are differently shaped by interactions between burden of treatment and social inequalities, and (c) provide a platform for responsive service design.

### Objectives

***Identification of studies***

We will systematically review and search for qualitative studies of the lived experience of three kinds of condition: long-term conditions associated with significant disability (Parkinson’s disease and schizophrenia); serious relapsing remitting disease (Inflammatory Bowel Disease, bipolar disorder); rapidly progressing acute disease (brain cancer, early onset dementia).

***Qualitative analysis***

Within materials included in the review, we will; (a) identify the work of service user and caregiver engagement and participation in self-care, health and social care; (b) characterise how these are differently shaped by interactions between burden of treatment (negotiated and assumed obligations), and social inequalities, and (c) understand the elements of these that could contribute to responsive service design.

***Theoretical development***

We will develop from this literature review (a) a taxonomy of service user and caregiver work associated with lived experiences of different condition types and trajectories, (b) a taxonomy of theoretical constructs that explain interactions between condition types and trajectories, service contexts and social inequalities and (c) a translational framework to support the development and implementation of new, person centred models of care for service users and caregivers. 

## Research plan

### Design and conceptual framework

Following the procedures we developed for the EXPERTS I study ^32, 39^ we will perform a theory-informed synthesis of qualitative studies. The study will employ conventional ‘manual’ qualitative analysis ^40^ and we will add a new dimension to our work by using Leximancer ^®^ software. This will enable us to perform text mining across the data set. In both text mining and manual analysis, we will explore the extent to which constructs are present across the qualitative data set, or whether they are concentrated around particular index conditions or disease trajectories.

***Linking healthcare constructs with social care literature***

An important theoretical and methodological problem in this review is synthesising research literature from different fields in which different technical vocabularies and theoretical constructs are employed. We expect to find differences in the ways that health and social care researchers identify, characterise and explain key constructs. We need to understand this better as we produce a coding manual. As our coding frame is developed, we will seek advice from professionals and service users across the health and social care field. Beginning with our oversight group, we will use a variety of techniques to identify useful discussants, and we will also use social media channels to identify experts by experience and academic experts to contribute to this process.

***Development of a coding manual for the review***

Because we will be using Leximancer software that performs semantic and relational searches, we need to create at the outset a coding manual or lexicon for the whole study. This will involve developing a set of theory-informed terms (e.g. Strauss *et al.*’s concept of articulation work ^8^, or Vassilev *et al.*’s concept of collective efficacy ^14^) that can be translated into everyday language and then used to search text. To do this, we will draw on three bodies of theory:

(i) Core concepts in the writings of Anselm Strauss and colleagues in the US that has set out interactionist models of ‘work’ as an integral element of the lived experience of health and illness ^8, 41, 42^, and status passage theory as a way of understanding the ways in which these lived experiences lead to service user and caregiver being identified as particular kinds of participant in these processes.
(ii) Our contributions to theories of service user work and capacity that have included Normalisation Process Theory ^43, 44^, and Burden of Treatment Theory ^1^ and participation in the development of the Cumulative Complexity Model ^15^. These have specified particular configurations of normative expectations of service users and caregivers (negotiated and assumed obligations), patterns of workload and capacity and their consequences.
(iii) Structural theories of social inequalities ^45^, networks ^46^, and social capital ^47^. These provide a set of fundamental conceptual building blocks for modelling important the social context of burden of treatment for service users and caregivers.

We will draw together key concepts from these theoretical frameworks in a taxonomy that will form the basis of a coding manual that will define what we are looking for in the manual analysis of papers included in the review. They will also form the core of a lexicon that can be used to define semantic and relational searches in text mining.

### Systematic literature searches

The protocol for the review is made publicly available on PROSPERO ( ID CRD42020224787). Literature searching will be contracted out to York Health Economics Consortium (YHEC), who have an internationally acknowledged team of information specialists. In collaboration with them, we will develop a search strategy for systematic searches of the following databases: Social Care Online, Science, Social Science and Arts and Humanities Citation Indices (Web of Science); CINAHL (EBSCO Host); EMBASE (Ovid); MEDLINE (Ovid); PsycINFO (Ovid); Scopus; PubMed. Search development is likely to be an iterative process given the complexity of the topic. The search will be peer reviewed by a second information specialist and the performance of the strategy in finding known relevant studies will be tested.

YHEC will run searches and conduct de-duplication of citations, providing Endnote database files of citations for screening. The search strategy is likely to use a multi-stranded approach using several different conceptual combinations, reflecting the fact that relevant records may not be consistently described. The social care literature may use different terms to describe phenomena of interest to the health care field. An example of the multi-stranded approach employed in this work might be: (1) (Index conditions OR generic terms for long term conditions) AND experience terms; (2) (Index conditions OR generic long term conditions terms) AND concept of service users AND qualitative research terms; (3) 1 OR 2; limit 3 to English language; (4) limit 4 to records including abstracts; (5) limit 5 to records published in the year 2010 onwards; (6) NOT (editorials OR comments etc.). A complementary search strategy will be developed to locate studies that pertain to informal carers. Further strands (i.e., conceptual combinations) are likely to be identified during strategy development.

### Inclusion criteria

We will ***include*** reports that meet all of the following general criteria, but we will tailor or stratify the inclusion criteria to meet specific features of index conditions.

- **Participants.** people aged >18 years; diagnosed with Parkinson’s disease, Inflammatory Bowel Disease, brain cancer, early onset dementia, bipolar disorder, schizophrenia, and their caregivers.
- **Reports.** results of qualitative studies of service users’ or carers’ accounts of the lived experiences of eligible conditions; their interactions with health professionals, healthcare provider organisations, treatment settings, technologies and regimens of care and self-care; and the social and economic contexts in which experiences of illness and care are set.
- **Study designs.** primary qualitative studies using semi-structured and unstructured interviews; primary qualitative studies using participant or non-participant observation; systematic reviews of qualitative studies, qualitative meta-syntheses and meta-ethnographies.
- **Settings.** Studies of illness experiences within self-care programmes, healthcare systems, and social care systems.
- **Date of publication.** Because there have been important changes in the organisation of care (and especially self-care) in recent years, we will restrict eligible studies to those published between 1 January 2010 and 31 March 2022.
- **Language.** English.

### Exclusion criteria

We will ***exclude*** reports which do not report the results of qualitative research with service users or carers; reports of treatment effectiveness, for example, RCTs, where the focus is on the treatment effect rather than the service user's or carer's experience; reports of healthcare organisation or delivery which are not focused on service user's or carer's experience; and editorials, notes, letters and case reports.

### Screening

Searches are likely to generate a very large number of records and so first stage screening will eliminate those that are obviously irrelevant, such as notes, comments, editorials, non-systematic reviews, RCTs and studies in diseases that are not eligible. Second stage screening will start with an assessment of relevance of citations and abstracts by two reviewers independently. Any studies which are eligible (i.e. they meet the criteria set out above) or which may be eligible (i.e. where the content is unclear, or reviewers disagree) will be obtained in full text. If agreement about inclusion cannot be reached, we will call on an independent assessor to act as final arbiter. Full text papers will be screened by two reviewers independently. Papers selected for inclusion will be stored as PDF files in secure Endnote Libraries with automatic back up. Records excluded based on assessment of full text will be listed in an excluded studies table with a brief reason for exclusion.

### Quality assessment of eligible articles

There are many proposed sets or reporting criteria for qualitative studies. We will use the CASP ^48^ checklist to assess the quality of qualitative research proposals and papers. It provides clear criteria for identifying high-quality reports. However, since there is no universally accepted reporting standard for qualitative studies, CASP can only guide decision-making on eligibility for inclusion. This is especially important because we will be drawing on bodies of literature (e.g., social work and social care) that may have different disciplinary criteria for reporting. Reports that provide insufficient information about sample, question, method and setting will be excluded from the review. In addition to the CASP checklist items, we have included an assessment of relevance for our review in regard to mention of illness trajectories, social inequalities, burden of treatment, and stigma.

### Data extraction

We will use two approaches to data extraction:

- **Manual Data Extraction.** We will undertake open and theory-informed coding. In theory-informed coding we will use the taxonomy of theoretical constructs described in the ‘Design and conceptual framework’ section above. We will design a data extraction instrument, develop a coding strategy, and write a coding framework and manual ^40^. As in our earlier reviews, we will test and refine this in a preliminary analysis of a sample of papers. This coding framework will be integrated into NVivo 12 ^®^ Software. Researchers will then independently read and code papers, recording the results of this work in NVIVO files. Where disagreements about coding occur, they will be arbitrated by a third member of the team. A preliminary coding frame is included in Table 1.
- **Text mining.** Leximancer ^®^ is a text mining application that is used in studies of consumer behaviour and marketing and in systematic reviewing in computer science and related fields ^49^. It has been little used in health services research. We will integrate our coding framework or lexicon in Leximancer’s pre-processing module. We will then run this across the whole data set of included papers ^50^. Leximancer uses semantic and relational algorithms to search for frequencies of groups of terms and for associations between them ^49^, and it produces maps and models of the relationships between them ^51^. It thus identifies empirical regularities in natural language data, and suggests ways in which they are connected ^52^. We will investigate these: searches will be informed by terms from our own coding framework as well as its own open coding of a qualitative data set, we expect that it will suggest new concept labels, and new patterns of lexical association between them ^53^.

**Table 1.** Preliminary coding frame.

Topic (1 ^st^ Order) Code	Specific (2 ^nd^ Order) Code
**1. Structural inequalities.** Are structural disadvantages identified in the paper and are aspects of their role in shaping patient and caregiver burdens and trajectories of illness characterised and explained?	**1.1 Intersectional disadvantage** (age, ethnicity, sex/gender, sexual orientation) **1.2 Material disadvantage** (social class, housing class, educational attainment, employment, income, pensions, social security/health insurance, access to internet)
**2. Spatial inequalities.** Are spatial inequalities identified in the paper, and are aspects of their role in shaping patient and caregiver burdens and trajectories of illness characterised and explained?	**2.1 Spatial relations** (spatial distribution of services, transport links, proximity to formal and informal support) **2.2 System boundaries** (organisation of services, professional divisions of labour, hierarchies of care/caregiving)
**3. Service inequalities.** Are service inequalities identified in the paper, and are aspects of their role in shaping patient and caregiver burdens and trajectories of illness characterised and explained?	**3.1 Category disadvantage** (stigmatised disease, stigmatised social group, health/social care boundary) **3.2 Modes of delivery** (algorithms/triage systems, access to specialist services, in-person vs telecare, web-enabled services)
**4. Interactional inequalities.** Are interactional inequalities identified in the paper, and are aspects of their role in shaping patient and caregiver burdens and trajectories of illness characterised and explained	**4.1 Provider behaviour** (acknowledgment of expertise, distribution of expert knowledge and practice, interaction opportunities, interaction quality, communications skills) **4.2 System responsiveness** (coordination between services and specialisms, responsiveness to crisis, relational quality, continuity of care)
**5. The affective self** Are aspects of **affect** identified in the paper, and how are these related to enacting, negotiating and navigating experiences of illness and care?	**5.1 Feelings** (of anxiety, fear, guilt, shame, denial, isolation) **5.2 Loss** (of self-esteem, self-worth, loss of intimacy, feelings of dependence & loss of independence, loss of sense of time & place) **5.3 Distress**, (fear-avoidance, decisional conflict, changing self-identity, implications of diagnosis & prognosis)
**6. Self-identity:** Are aspects of self-identity discussed in the paper, and how are these related to enacting, negotiating and navigating experiences of illness and care?	**6.1 Illness identity** & personal meanings, control & disclosure of information about self & illness, tolerance of disruption **6.2 Biographical disruption** & erosion, social withdrawal, felt & enacted stigma, changing personal and lifeworld priorities, loss of employment and status **6.3 Stigma** (internalised [felt] stigma, externalised [enacted] stigma) **6.4 Resilience:** tolerance of distress and **disruption** Problem- solving, loss of social competence & functioning
**7. Making sense of the self.** Are aspects of personal sense- making identified in the paper, and how are these related to enacting, negotiating and navigating experiences of illness and care??	**7.1 Symptoms** (recognition & awareness, knowledge of disease processes & outcomes, self-monitoring, self-management strategies) **7.2 Restrictions** (Physical and psychological pain & discomfort; restrictions on diet, movement & social interaction, others' disbeliefs and misperceptions of symptoms & disease processes) **7.3 Disease progression** (unpredictable relapse-remission cycles, uncertainty about capacity to manage disease)
**8. Making sense of others.** Are aspects of distributed sense- making identified in the paper, and how are these related to enacting, negotiating and navigating experiences of illness and care?	**8.1 Roles** (domestic contributions, personal and shared decisions, emotional & relational solidarity, others monitoring self and health, competing priorities of other, crises readiness for crises) **8.2 Others** (lack of knowledge, domestic rountines, disorganisation, dependency, interpersonal & decisional conflict, integration of illness identity into family relations, relations with significant others) **8.3 Social networks** (informal social networks & network formation, restoration of social capital, collaborations around care & self-care, other sources of social support, sources of resilience.
**9. Administrative burden.** Are aspects of administrative burden identified in the paper, and how do these relate to the ways that service users and caregivers enact, negotiate, and navigate their formal relations with health/care providers?	**9.1 Competence,** social skill, securing cooperation, coordination of care, financial acumen. **9.2 Organisational behaviour:** terminology and **meanings**, appointments, tests, communication between service providers, financial support. **9.3 Costs of illness:** expenditure, **access** to services, service organisation, informational & relational fragmentation of systems.
**10. Delegated care work.** Are aspects of delegated care work identified in the paper, and how do these relate to the ways that service users and caregivers enact, negotiate, and navigate health/care knowledge and practice at home?	**10.1 Responsibility**, prudence, multiple medications, testing & monitoring equipment, pain control, supportive equipment, mobility aids **10.2 Workload:** medical terminology and meanings, temporal and cognitive **demands** of care, supply of medication/ equipment, provider role & obligations, patient/caregiver role a& obligations **10.3 Task/cost shifting**, self-care/care skills, knowledge & practice, lifestyle changes, dietary **changes**
**11. Help-seeking:** Are aspects of help-seeking identified in the paper, and how do these relate to the ways that service users and caregivers enact, negotiate, and navigate access to care when help is needed?	**11.1 Resourcefulness**, appointments/triage systems, access to emergency care, interactions with providers **11.2 Candidacy**, warrantability access to service providers, investigations, hospitalization (voluntary/involuntary), conflict with providers **11.3 Fragmentation:** spatial and temporal **fragmentation** of services
**12. Managing the consequences of disease.** Are consequences of disease identified in the paper, and how do these relate to the ways that service users and caregivers enact, negotiate, and navigate care pathways, decisions about treatment, questions about mental capacity/cognitive deficits?	**12.1 Cognitive authority & sick role**, medication decisions, requesting/refusing treatment, maintenance of social networks **12.2 Loss of control**, medication side effects, treatment choices, treatment escalation, advance care plans, participation of caregivers/others, palliation, provider expectations of patients/caregivers, increasing workload, diminishing capacity **12.3 Care pathways**, professional/service boundaries, pathophysiological deterioration, status passage

### Data analysis

Qualitative data analysis will follow the three-stage process that we have previously used to develop a robust conceptual model. Our approach to data analysis is abductive ^54^ in perspective, informed by attribution theory ^55^. We are searching for different kinds of empirical regularities in natural language data, and for the ways that these include characterisation and explanation of relevant phenomena, rather than for the de novo emergent themes that would be discovered in an inductive phenomenological or grounded theory study.

***Taxonomy-building***

The first phase of analysis is descriptive. Using both manual and text mining applications we will produce taxonomies of

(I) the work of service users and caregivers in participating and engaging with the expectations of self-care, health and social care providers;
(II) the work of negotiating and interacting with health and social care providers and professionals;
(III) the shaping effects of social inequalities and structural disadvantages.
(IV) The content of these taxonomies will vary between index conditions and disease trajectories, but they will produce large numbers of potential taxa. To prepare for modelling, we will identify and eliminate redundancies and duplicates amongst these.

***Characterisation and modelling***

We will characterise and compare patterns of taxa and constructs for six index conditions; three disease trajectories; three service contexts, and one universal set of constructs which appear in all index conditions and all disease trajectories. In the early phases of modelling, we will therefore assess the relative significance and the degree of universality of any particular construct. We will also assess the position and role of constructs in relation to each other. For example, these constructs may characterise preconditions, resources, relationships, or endpoints. We will then sift and sort constructs, writing them as context-independent propositions that are linked to four general categories of work revealed in the literature over five decades: being (negotiating experience and identity); changing (managing status passage and biographical disruption); relating (participating in interactions within healthcare provision and informal social networks); and doing (performing health behaviours and enacting healthcare technologies and self-care practices). We will continue this until we reach the most parsimonious possible model of interactions between constructs.

***Construct validation***

The final stage of analysis is to link context dependent propositions together in a summary statement that characterises and explains the operation of the model and its implications in ways that can be easily understood. The completed analysis and summary statement will be presented to Patient and Public Involvement (PPI) reference groups and to service user and caregiver advocacy groups, and to a reference group that includes social and health care practitioners and researchers. At this stage, we will also explore scenarios around workload reduction strategies and person-centered service design for people with complex health problems. This is to ensure that the model's constructs and propositions have (i) face validity for people who experience health and social care provision, and for other practitioners and researchers in the field, and (ii) that they can inform supportive interventions that are practically workable across a range of treatment modalities, service organisation and delivery.

## Engaging service users, professionals and policy makers

### Practitioners, NHS and Social Care Managers

In the construct validation and translation phase of the study we will undertake dissemination and engagement activities with stakeholders to explore ways in which the constructs developed within the review might inform supportive interventions that are practically workable in reconfigurations of treatment modalities, service organisation and delivery. We will use ARC communications services to disseminate these results to NHS Integrated Care Systems, Sustainability and Transformation Partnerships, Clinical Commissioning Groups, Local Authorities, private and third sector Service Providers in health and social care.

### Service users, caregivers and their advocates

We will engage with service users and caregivers, and their advocates and work with these stakeholders to co-create digital materials and animations, which we will publish on the web using Instagram and Youtube, and also through our interactions with www.patientrevolution.org.

### Policy-makers

We will take full advantage of opportunities for face-to-face interaction, analogue and digital media to promote the results of this review. We will develop and implement a robust and ambitious strategy to communicate results of this work. To engage policy-makers at a national level we will work to identify and engage with key national policy-makers and influencers, provide them with key policy briefings (in both web and hard copy form).

### Research community

Dissemination to the research community will be through open access journal articles, conference presentations, and seminars. In addition to the final report to be published as a peer-review journal monograph in *Health Services and Delivery Research,* we will publish our protocol in *NIHR Open*; report on our theoretical framework; methodological aspects of text mining in qualitative systematic reviews; and comparative models of service user and caregiver workload and capacity in physical and mental health problems. We will also propose a workload reduction model for service user and caregiver burden in complex disease trajectories.

## Ethics

This is a literature review and does not involve research on human subjects. Ethics Committee approval is therefore not necessary.

## Service user and public involvement

This proposal stems from meetings held with members of the PPI reference group of the Complexity, Service user Experience and Organisational Behaviour research theme (jointly led by May and Richardson) of NIHR CLAHRC Wessex (now superseded by NIHR ARC Wessex) between 2015 and 2019. Members of that group consistently pointed to the complex, time-consuming, and sometimes arduous work that they needed to do to effectively engage with NHS services. They pointed to the ways in which NHS services were often fragmented and uncoordinated, that they often experienced care pathways as arbitrary sequences of interactions, and that they struggled to make sense of the processes of care within which they were involved. In this proposed study, the lessons of that earlier PPI input have been taken on board.

## Anticipated impact and dissemination

Through our detailed description of the work undertaken by service users and caregivers to engage and participate in self-care, health and social care, practitioners and researchers will be better placed to understand structural factors that shape treatment burden and affect service user activation across a range of different illness trajectories. Additional impact of this research will be the identification of promising targets for service redesign, and for policy restructuring. Dissemination strategies include the promotion of workload reduction models to support service users and caregivers and their advocates, and policy briefs and peer-reviewed reports for practitioners, policy makers, and researchers. 

## Data Availability

No data associated with this article.
