# Supplementary material for: EXPERTS II - How are patient and caregiver participation in health and social care shaped by experienced burden of treatment and social inequalities? Protocol for a qualitative synthesis
Source: NIHR Open Res. 2023 Jun 16;3:31. [Version 1] doi: 10.3310/nihropenres.13411.1 (PMC10593344; doi:10.3310/nihropenres.13411.1)
Supplement: Peer review history

# Reviewer report: Reviewer response for version 1

**DOI:** 10.3310/nihropenres.14544.r30046

**Published:** 2023-08-23

**Authors:** Gloria Wong

**Affiliations:** 1 The University of Hong Kong, Hong Kong, Hong Kong

This research aims to investigate service user/carer workload and its interaction with social inequalities to inform service redesign. This aim, if achieved, can be impactful. The method is a combined manual and text-mining approach to the qualitative synthesis of qualitative studies, of users/carers' accounts on a range of physical and mental health conditions categorised into three predefined trajectories. The protocol is generally well-written; however, due to this work's scope and importance, I would appreciate it if the authors could clarify some conceptual issues at this stage. 

1. As the aim is to inform service redesign for reducing user/carer workload, the rationale for selecting illness trajectories as the key framework for analysing the data deserves more explanation, as to its theoretical relevance to user/carer workload and service organisation compared with other illness characteristics (e.g., onset age, mental capacity, comorbidity). Do the authors expect that the findings by illness trajectory categories will be applicable to all illnesses with similar trajectories, and services be redesigned according to their expected trajectory? This is an important question to allow readers to understand how the research method matches its aim. Once this is clarified, it would also be helpful to clarify in the 'Definition of trajectories' section the actual criteria for fitting diagnostic categories into these three trajectories, given the possibility of overlap between these categories (schizophrenia can be considered a serious relapsing-remitting disease) and within diagnoses (some people with bipolar disorders and early-onset dementia can experience long-term disability, some people with schizophrenia have little disability).
2. The use of text mining appears to be a useful way to identify new concepts to complement a manual approach. It would be helpful if the authors could explain the rationale for adopting a more top-down, theory-informed approach to developing the coding manual for text mining, and how might that be different or similar to their manual data extraction approach, which is also theory-informed. The considerations for not adopting more grounded or bottom-up approaches in the data analysis could be provided. Please also clarify the data to be included in the text mining: would this involve only verbatim quotes from users/carers provided in the included papers? Would the information from CASP be used to select data or interpret results from the text mining? Given that the data for analysis in this study is secondary, the authors may wish to clarify whether their findings will reflect user/carers' actual experience of workload, or the prevailing themes accepted by researchers/journals only.
3. In the preliminary coding frame, the logical links with the research questions and aims could be made more explicit. There seems to be a focus on the negative attributes of existing services (e.g., fragmentation) and user/carers' experience (e.g., negative feelings); some key constructs in the research questions (e.g., capacity) are not included; yet other constructs (e.g., self-identity) not defined earlier in this paper included. Notwithstanding the fact that the final codes are to be developed, it would be helpful to explain if the authors would use any strategies to ensure the relevance of the findings to the research aim, given the amount of information and almost infinite possible ways of coding and organising these codes, and the need to derive constructive conclusions for policymakers and practitioners. 
4. Throughout the protocol, the authors are clear about the ultimate goal, which is to promote workload reduction model. I would like to learn from the authors whether it is their intention to be focusing narrowly on workload reduction from a user/carer-only perspective, or do they mean reduced workload while balancing the need of maintaining health, and others' needs from a societal perspective. For example, there will likely be studies examining users/carers' burden, workload, negative feelings, loss, and distress involved in compulsory hospital admission in schizophrenia. Such practices continue to exist with numerous discussions balancing the risks and benefits to others and to the person (including the possibility of regaining sufficient health, capacity and thus control after a period of significant burden), and how to improve the practice on balance. While both are acceptable perspectives, a clear positioning of this research will help readers to understand its roles in guiding policy and practice.

Is the study design appropriate for the research question?

Yes

Is the rationale for, and objectives of, the study clearly described?

Yes

Are sufficient details of the methods provided to allow replication by others?

Partly

Are the datasets clearly presented in a useable and accessible format?

Partly

Reviewer Expertise:

Schizophrenia and other psychotic disorders, dementia, mental health services, long-term care, person-centred care

I confirm that I have read this submission and believe that I have an appropriate level of expertise to confirm that it is of an acceptable scientific standard, however I have significant reservations, as outlined above.

# Reviewer report: Reviewer response for version 1

**DOI:** 10.3310/nihropenres.14544.r30051

**Published:** 2023-08-08

**Authors:** Phyllis Dako-Gyeke

**Affiliations:** 1 University of Ghana, Accra, Greater Accra Region, Ghana

This protocol seeks to conduct a qualitative synthesis involving the construction of a theory informed coding manual, analysis of papers leading to the construction of a taxonomy, and design of a model. The paper is very rich in content, however authors may consider the following comments to improve on the methodology:

1. Their "Definition of participants" seems to be very crowded with information, which makes it difficult for readers to follow. It will be good to keep it simple and straightforward.
2. Consider defining other terminologies in the title such as " "treatment burden" and "social inequalities."
3. The authors are not clear on the scope of work they are focusing on, whether globally, nationally, high income contexts, low-to-middle income contexts, etc. This is important as the relevance of the developed models will be determined by the context within which the various studies were conducted. I believe the issues relating to negotiations and trajectories will be different depending on context. Also, inequalities could be measured at both macro or micro levels. Either authors give a good explanation or state it as a limitation. 
4. Towards the end the authors allude to the fact that they will be looking at both papers and proposals. Not sure how the review of proposals will be useful to this process. 

Is the study design appropriate for the research question?

Yes

Is the rationale for, and objectives of, the study clearly described?

Yes

Are sufficient details of the methods provided to allow replication by others?

Partly

Are the datasets clearly presented in a useable and accessible format?

Not applicable

Reviewer Expertise:

Implementation research, qualitative methodologies

I confirm that I have read this submission and believe that I have an appropriate level of expertise to confirm that it is of an acceptable scientific standard.

# Reviewer report: Reviewer response for version 1

**DOI:** 10.3310/nihropenres.14544.r29923

**Published:** 2023-07-27

**Authors:** Helen Snooks

**Affiliations:** 1 Swansea University, Swansea, Wales, UK

The paper could be written in plainer English - especially in the Plain English Summary, but actually throughout - I had to read the abstract and PES several times before I really understood that this is about the experience of patients/service users as well as carers - "workload" and "trajectory" are unfamiliar concepts in this context and could be explained or perhaps rephrased.

 Definition of participants - nicely explained use of service user throughout paper (but not as review term).

 Other definitions also useful - good to present this at the outset of the protocol.

 It felt to me that there may be too many papers that meet the inclusion criteria for this review to be feasible - do the authors have a preliminary idea of the volume of papers that they may need to include - and maybe a plan  B - could they include a short section on this in the protocol paper?

Is the study design appropriate for the research question?

Yes

Is the rationale for, and objectives of, the study clearly described?

Yes

Are sufficient details of the methods provided to allow replication by others?

Yes

Are the datasets clearly presented in a useable and accessible format?

Yes

Reviewer Expertise:

Health Services Research, mixed methods

I confirm that I have read this submission and believe that I have an appropriate level of expertise to confirm that it is of an acceptable scientific standard, however I have significant reservations, as outlined above.
